# Regulation of photosynthetic material production by inter-root microbial extinction and metabolic pathways in sorghum under different nitrogen application patterns

**DOI:** 10.1038/s41598-022-10969-4

**Published:** 2022-04-26

**Authors:** Zhang Fei, Jiaxu Wang, Kuangye Zhang, Han Wu, Fulai Ke, Youhou Duan, Yanqiu Wang, Jianqiu Zou, Kai Zhu, Zhipeng Zhang, Feng Lu, Hongtao Zou

**Affiliations:** 1 Sorghum Institute, Liaoning Academy of Agricultural Sciences, Dongling Road 84 District, Shenyang, 110161 Liaoning China; 2 Soil and Resource Environment Institute, Shenyang Agricultural University, Dongling Road 120 District, Shenyang, 110161 Liaoning China

**Keywords:** Physiology, Plant sciences

## Abstract

The development of nitrogen fertilizer green and efficient application technology by exploring the mechanism of efficient sorghum N use is important for sustainable development of sorghum industry as well as barren marginal land development and utilization. This study was conducted in 2018, 2019, and 2020 at Shenyang, China, using the nitrogen-efficient sorghum variety Liaonian No. 3 as material. The correlation between soil microbial species, diversity, and metabolic pathways with photosynthetic parameters and yield traits was analyzed to elucidate the mechanisms of nitrogen utilization and photosynthetic material production in sorghum under four fertilizer application patterns. The results showed that 17 populations of soil inter-root microorganisms were active in the organic fertilizer + 0 kg per hm^2^ of nitrogen (N0Y) model, and the abundance of two key populations, Comamonadaceae and Ellin5301, was significantly increased. Soil microorganisms regulated sorghum growth mainly through 30 pathways, focus including ko00540, ko00471, ko00072 and ko00550, of which ko02030 (Bacterial chemotaxis) and ko00072 (Synthesis and degradation of ketone bodies) played the most critical role. The functional analysis of soil microbial populations revealed that N0Y fertilizer model significantly reduced the intracellular trafficking, secretion. In addition, vesicular transport of microorganisms, amino acid transport and metabolism and nucleotide transport and metabolism played a key role in the regulation of population function. Overall, the N0Y model of N-efficient sorghum can achieve high levels of photosynthetic material production and higher yield formation through regulation of population activities and metabolic pathways of loamy microorganisms, resulting in reduced chemical N application and efficient green production of sorghum.

## Introduction

Sorghum (*Sorghum bicolor* L. Moench) is the fifth largest crop in the world and a crop that combines the resistance characteristics involves drought, salt, flooding, low temperature, and growth on barren lands, of which plays an important role in the world agricultural production and marginal barren lands exploitation^1,2^. After years of exploration and practice, sorghum has become the preferred crops for barren lands due to its excellent barrenness tolerance during pollination, it requires much less fertilizer nutrients than other dry-land crops such as maize and wheat^3^. It has been suggested that external supplementation of nitrogen supply is an important means of promoting high sorghum yields^4^, and it has also been suggested that excess nitrogen supply can limit the growth and development of sorghum thereby affecting the development of higher yields^5,6^. Therefore, nitrogen supplementation for different sorghum varieties has become a critical issue for sorghum industry development.

Fertilizer application has been shown to play a supportive role in increasing crop yields and improving quality^7,8^. The large-scale use of chemical synthetic inorganic fertilizers in agricultural production as early as the middle of the twentieth century contributed significantly to the growth of global crop yields^9^. However, at the same time, the perennial application of chemical fertilizers has brought a potential crisis to the ecological environment, and in some areas, excessive fertilizer application have led to serious environmental and land pollution and waste of resources^10,11^. With the increasing crop yield and fertilizer application, the impact of fertilizer application on the ecological environment of farmland has received much attention from researchers, especially the quality of soil environment and quality and safety of agricultural products caused by fertilizer application has attracted wide attention worldwide^12,13^. In this context, scholars have carried out experimental research on chemical fertilizer alternative technology, straw return, precision fertilizer application and formula fertilizer application, and agreed that chemical fertilizer alternative technology is the most effective, and the study shows that the combination of chemical fertilizer and organic fertilizer is an important means to solve the excessive use of chemical fertilizer, improve the soil environment and enhance crop yield^14,15^.

The richest habitat for biodiversity on earth is the soil, which is rich in organic and inorganic material that provides habitat for various biological communities^16,17^. Soil microorganisms are involved in ecological processes such as nutrient decomposition and supply, and they are an essential component of growth and yield formation^18^. Soil microorganisms use various nutrients in the ecosystem material cycle to fulfill their own growth needs while continuously improving the soil environment. In addition, the application of chemical and organic fertilizers in agricultural production has an impact on the physical and chemical properties of the soil, as well as a certain degree of change in the soil biological environment, which in turn affects crop growth^19,20^.

In order to explore the efficient ratio pattern of organic and chemical fertilizers for sorghum, the optimal integration technology of livestock manure and inorganic chemical fertilizers was conducted by sorghum as experimental material in this context. And the aim is to reveal the causes of high yield formation of nitrogen utilization in nitrogen efficient sorghum from the perspective of root microbial and colony regulation, to elucidate the intrinsic mechanism of photosynthetic regulation and root secretion regulation in nitrogen efficient sorghum, and to integrate sorghum nitrogen fertilizer efficient utilization and organic manure optimal integration technology to provide technical support for green and efficient sorghum production.

## Materials and methods

### Test material and location

The experiment was conducted using a nitrogen-efficient sorghum variety (*Liaonian No. 3*) selected from 628 varieties, which has passed the appraisal of China National Appraisal Committee in 2008. It is widely grown in northeast and northwest China, with excellent brewing quality, high yield, multiple resistance to drought, flooding and salinity, and great potential for exploitation for crop gains.

The experiment was conducted at the experiment site of Liaoning Academy of Agricultural Sciences (Liaoning Shenyang, China, N41° 48′ 11.75″ E 123° 25′ 31.18) during the 2018, 2019 and 2020 growth seasons. The climatic factors of the sorghum growing season at the experimental site (May to September) were average day/night temperature 24.5/18.7C, relative humidity 45.36%, average rainfall 4.20 mm per day and actual full sunshine hours 6.80 per day. Soil nutrient status were total nitrogen 0.086%, total phosphorus 0.110%, total potassium 1.906%, hydrolytic nitrogen 61.3 mg per kg, effective phosphorus 13.63 mg per kg, effective potassium 102.6 mg per kg, pH 6.8.

### Experimental design

The experiment was set up with four fertilizer treatments, as it shown in Table 1. The dosage of phosphorus and potassium fertilizer was the same for all treatments, which was 75 kg per hm^2^ for phosphorus P_2_O_5_ and 75 kg per hm^2^ for potassium K_2_O. Organic fertilizer using chicken manure with 1.5% N content, which was spread into the soil at one time before sowing at a dosage of 6000 kg per hm^2^ and rototilled evenly with a rototiller at a depth of 25 cm from the soil surface. Nitrogen fertilizer was applied in two ways, including base fertilizer and chase fertilizer, and base fertilizer was applied at the same time with phosphorus and potassium fertilizer, and chase fertilizer was applied at the end of elongation stage, and the amount of base fertilizer and chase fertilizer of nitrogen fertilizer accounted for 1/2 of the total amount of fertilizer respectively.

**Table 1:** Abbreviations of different fertilizer treatments and corresponding fertilizer application methods.

Abbreviation for fertilizer treatment	Treatment patterns
N0W	No organic fertilizer + 0 kg per hm^2^ of nitrogen
N8W	No organic fertilizer + 120 kg per hm^2^ of nitrogen
N0Y	Organic fertilizer + 0 kg per hm^2^ of nitrogen
N8Y	Organic fertilizer + 120 kg per hm^2^ of nitrogen

The trial was conducted in a randomized group design with a plot row length of 6 m, a 10-row zone, a distance of 0.6 m, a plot area of 36m^2^, and three replications per year, with a total of nine replications in three years. The planting density was 12,000 plants per hm^2^, and the field disease, insect, weed and water management were equal to the local normal level.

### Soil sample collection and processing

Soil samples were collected from the roots of seedlings of each treatment at the flowering stage (80 days after sowing) for soil microbial diversity and colony population analysis. The soil sampling site was taken from the inter-root soil by pulling out the sorghum root and then shaking, three soil samples in close with the root were sampled for each sample as replicates, and the soil sampling weight was 200 g for each sample. After the soil samples were collected, they were stored at low temperature and stored in a − 80 °C refrigerator for later use.

### Extraction of total soil DNA and PCR amplification of 16S rRNA gene V4-V5 region

After the soil samples were thoroughly mixed, 0.5 g of soil were taken from each samples, and the OMEGA microbial genome extraction kit (OMEGA, USA) was used to DNA extraction. After DNA extraction, the purity Genomic DNA was tested using the following methods. Agarose gel electrophoresis for genomic DNA integrity: clear visible electrophoretic bands and no significant degradation. Nanodrop 2000 for genomic DNA quality: concentration ≥ 20 ng/μL, total ≥ 500 ng, OD260/280 = 1.8–2.0.

### PCR amplification of 16S rRNA gene V4–V5 region

Using the diluted genomic DNA (20 ng/μL) as the template, the primers in the V4-V5 region of the 16S rRNA gene were used for amplification. The primer sequences were 515F: 5′-GTGCCAGCMGCCGCGG-3′ and 907R: 5′-CCGTCAATTCMTTTRAGTTT-3′. PCR amplification system (25 μL): 5 × reaction buffer 5 μL, 5 × GC buffer 5 μL, dNTPs (100 mmol/L) 5 μL, 515F (10 μmol/L) 1 μL, 907R (10 μmol/L) 1 μL, DNA 2 μL, ddH_2_O 6 μL. Amplification parameters: pre-denaturation at 95 °C for 2 min, denaturation at 95 °C for 15 s, annealing at 55 °C for 30 s, extension at 72 °C for 30 s, 30 cycles; final extension at 72 °C for 5 min, incubation at 10 °C. After the amplified PCR product was detected positive by agarose electrophoresis, it was sent to Biotechnology Co., Ltd. for sequence sequencing and analysis using the Illumina Miseq high-throughput sequencing technology platform.

### Sequencing on Miseq

The libraries were sequenced using the Miseq platform with a 2 × 250 bp double-end sequencing strategy and subsequent bioinformatic analysis. Data analysis was performed using the software PICRUSt2 v2.2.0-b, R (v3.4.10) to obtain abundance prediction results for the COG (Clusters of orthologous groups of proteins) function of bacterial communities by PICRUST2. The COG database has two levels, with cog_level1 and cog_level2. The abundance prediction results of KEGG functions of bacterial communities were obtained by PICRUST2, and the functions were named by KO ID, which represented the specific functional genes, and then the information of 3 levels of metabolic pathways were obtained based on the information of KEGG database, and the abundance table of each level was obtained separately. The predicted abundance of the MetaCyc pathway in the bacterial community was obtained by PICRUST2. MetaCyc (https://metacyc.org/) contains pathways involving primary and secondary metabolism and related metabolites, reactions, enzymes and genes, which are classified according to the biological function of the pathway and the type of metabolites they produce or consume, and this system Display of MetaCyc secondary classification metabolic pathway diagram.

### Photosynthetic parameters

Photosynthetic parameters such as net photosynthesis (Pn), stomatal conductance (Gs), and transpiration rate (Tr) were determined at anthesis using a LI-6400 photosynthesis system (Li-Cor, Inc., Superior St., Lincoln, NE 68504). These measurements were carried out at local time from 9:30 AM to 12:00 AM. The measurements were repeated three times on the flag leaf (uppermost leaf) leaves of each plant. Means of three readings were used for statistical analysis.

### Chlorophyll content

The uppermost flag leaves of three plants were selected for sampling in each treatment at flowering stage, and the chlorophyll content was measured at the middle of the flag leaf by SPAD 502 chlorophyll meter.

### Yield and grain weight

At maturity, 8 rows × 0.5 row width × 4 m row length, total 12m^2^ including 200 plant of sorghum samples were taken from the middle of each plot by removing the side rows, and the seeds were weighed when they were air-dried to a moisture content of 14% to calculate the yield and finally converted to yield per hectare of area. In addition, three groups of seeds were taken for measurement, with 1000 seeds in each group to determine the seed weight.

### Data processing and analysis

Data were subjected to analysis of variance (ANOVA) in SAS (100 SAS Campus Dr., Cary, NC 27,513). The significance of main effects was determined at the 0.05 and 0.01 level with Fisher.

Soil microbial data analysis methods as belowed. First, the raw data were subjected to quality filtering and paired-end sequence ligation. The software Qiime and Mothur were used to filter the ligated sequences and remove chimeras. Then, OTU clustering was performed on the obtained high-quality sequences based on the similarity level of 97%, and species annotation was performed using the Greengene database. The Qiime software was used to draw the dilution curve, and the summary. Single command in the software Mothur was used to calculate four commonly used biodiversity indices: Chao, ACE, Shanon and Simpson indices. Principal component analysis (PCA) was performed on the taxonomy and species abundance at the genus level using the software R, and PCA diagrams were drawn. Statistical analysis of the community structure was carried out at each classification level to obtain the composition of the microbial community structure.

### Statement specifying

Our experimental research and field studies on sorghum including the collection of material all comply with relevant institutional, national, and international guidelines and legislation. We have stated that Ethics approval and consent to participate: No applicable.

## Results and analysis

### Number of soil microbial populations

**Figure 1:**
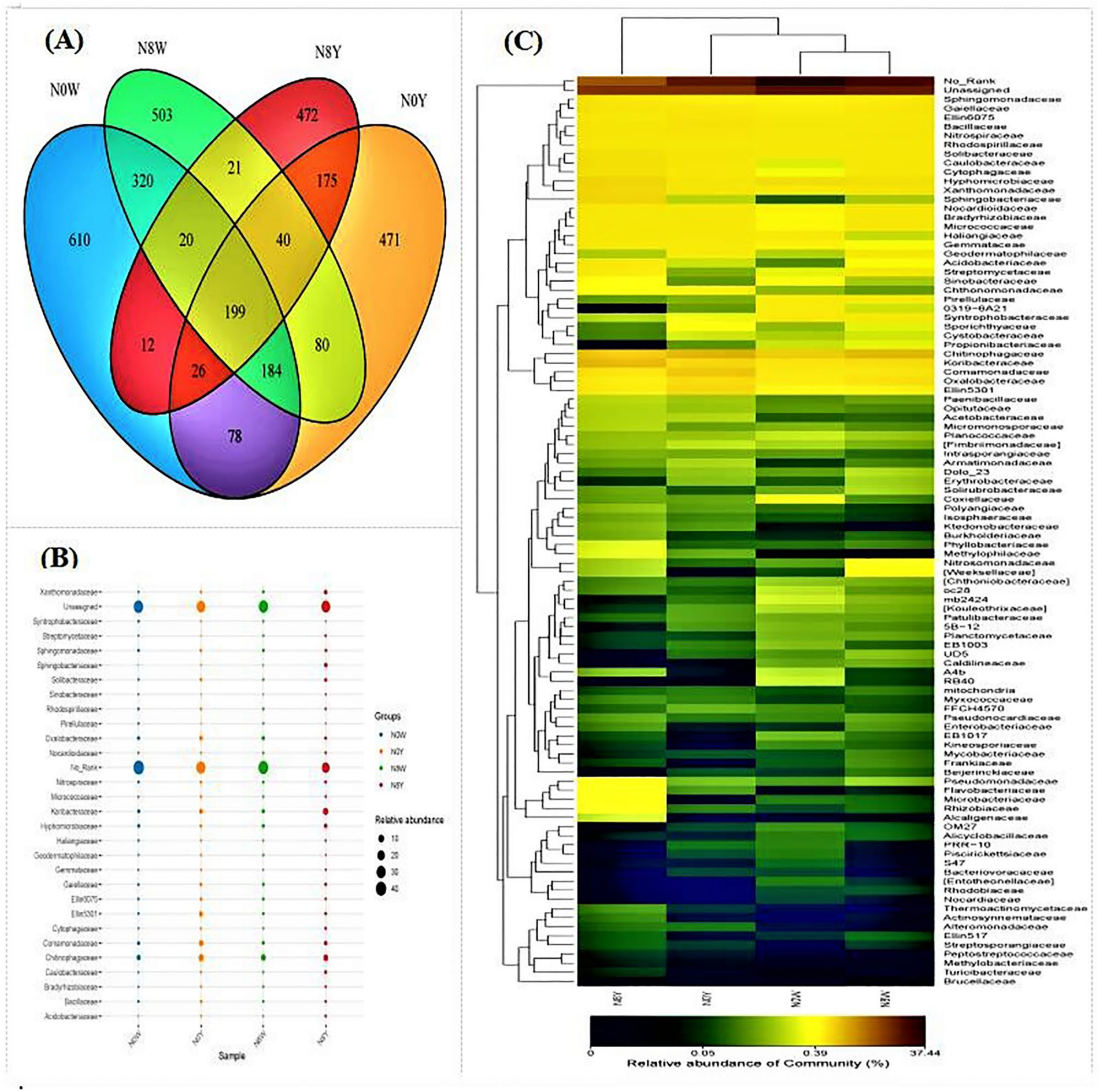
Changes in inter-root soil microbial populations of sorghum under different nitrogen conditions. (**A**) Venn diagram presumably of the microorganisms. (**B**) Bubble chart of soil microbial abundance distribution. (**C**) Cluster analysis heatmap of soil microbial.

The analysis of inter-root soil microbial characteristics under four different fertilizer treatments showed that fertilizer application had an effect on both soil microbial species and abundance (Fig. 1A–C). A total of 2603 populations were detected including 667, 650, 666 and 620 for N0W, N0Y, N8W and N8Y, respectively (Fig. 1A). With respect to the treatments with and without organic fertilizer, unique populations were detected at 610 and 471 for the N0W and N0Y treatments, respectively, with N0Y being 31.6% lower than N0W. This indicates that the number of unique microbial populations was reduced in the organic fertilizer application compared to the non-organic fertilizer treatment, and these populations are likely to be related to the effect of organic fertilizer. In contrast, 503 and 472 bacterial populations were detected under N8W and N8Y treatments, respectively, and N8Y was 6.2% lower than N8W, and under N0W and N8W, N8W and N0W were 6.1% lower than N8W. It indicates that these colonies are most likely related to N fertilizer application. In addition, 471 and 472 colonies were detected under N0Y and N8Y treatments, respectively, with very small differences in the number of colonies. The analysis of the 28 defined effective soil microbial populations (except unassigned and No rank) revealed that chitinophagaceae, oxalobacteraceae, and hyphomicrobiaceae three groups of microorganisms had higher relative abundance with organic fertilizers than without organic fertilizers (Fig. 1B). In addition, heat map clustering analysis revealed that the inmicroorganisms populations under different fertilizer application patterns were mainly concentrated in 2 taxa of which involving flavobacteriaceae, PRR-10, thermoactinomycetaceae, actinosynnemataceae, and alteromonadaceae, ellin517, streptosporangiaceae 6 groups of microflora, especially flavobacteriaceae showed significant differences between organic fertilizer application and organic fertilizer treatment, so it is likely to be the key factor in the microbial regulation of sorghum growth regulated by organic fertilizer (Fig. 1C).

### Diversity of soil microbial populations

**Figure 2:**
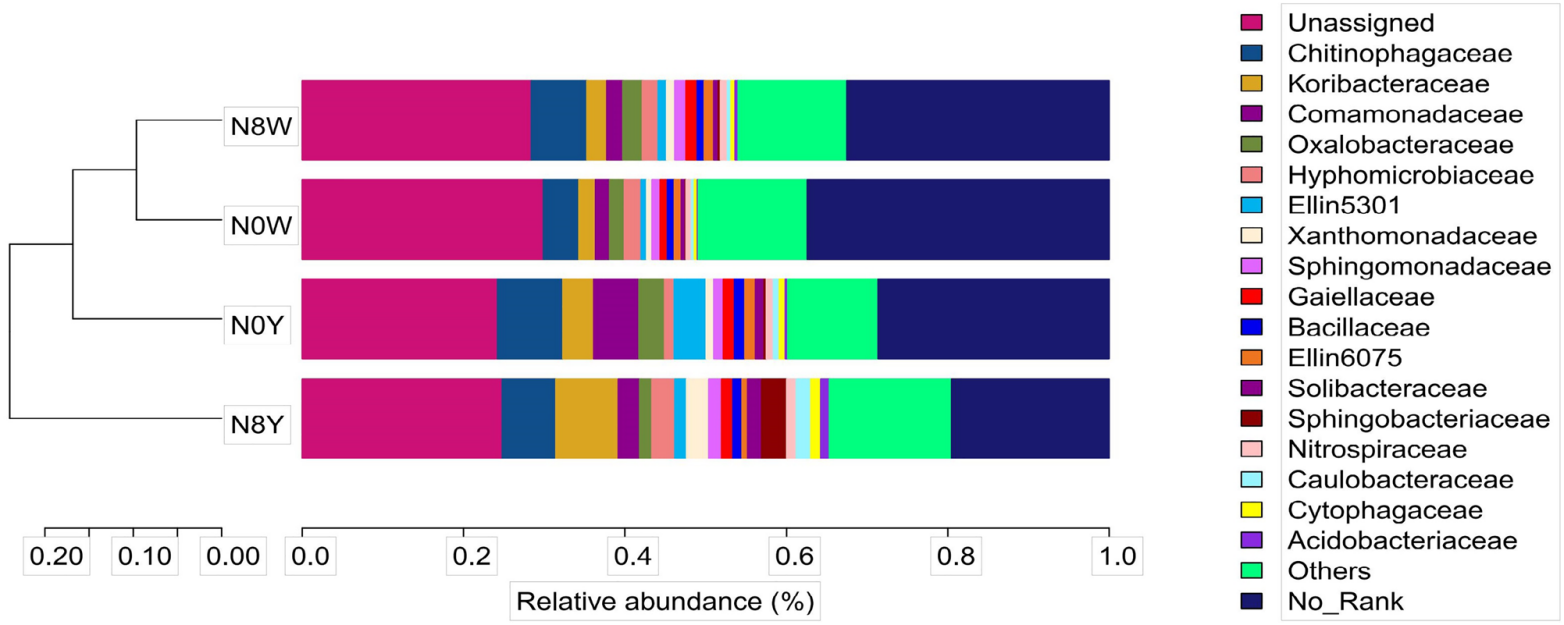
Analysis of relative abundance of soil microorganisms between sorghum roots under different nitrogen conditions.

The results of the study showed that there were differences in the relative abundance of bacterial populations in the four fertilizer patterns N0W, N0Y, N8W, and N8Y (Fig. 2). Overall, the abundance of soil microbial populations increased significantly after the application of organic fertilizers, mainly involving 17 species of chitinophagaceae, koribacteraceae, comamonadaceae, oxalobacteraceae, hyphomicrobiaceae, and xanthomonadaceae, etc (Fig. 2).

Chitinophagaceae, koribacteraceae, and comamonadaceae showed the most active variation. chitinophagaceae and koribacteraceae were higher than N0W under N0Y treatment, but the difference between N8Y and N8W was not obviously. The trends of comamonadaceae and ellin5301 among different nitrogen treatments were very similar to chitinophagaceae (Fig. 2). It indicates that the application of organic fertilizer at lower nitrogen supply levels significantly increased the relative abundance of chitinophagaceae, comamonadaceae, and ellin5301, but the changes were subtleties when the soil was supplied with a certain amount of nitrogen. In addition, koribacteraceae was higher under both N0Y and N8Y treatments than N0W and N8W, especially the N8Y was nearly threefold higher than N8W.

**Figure 3:**
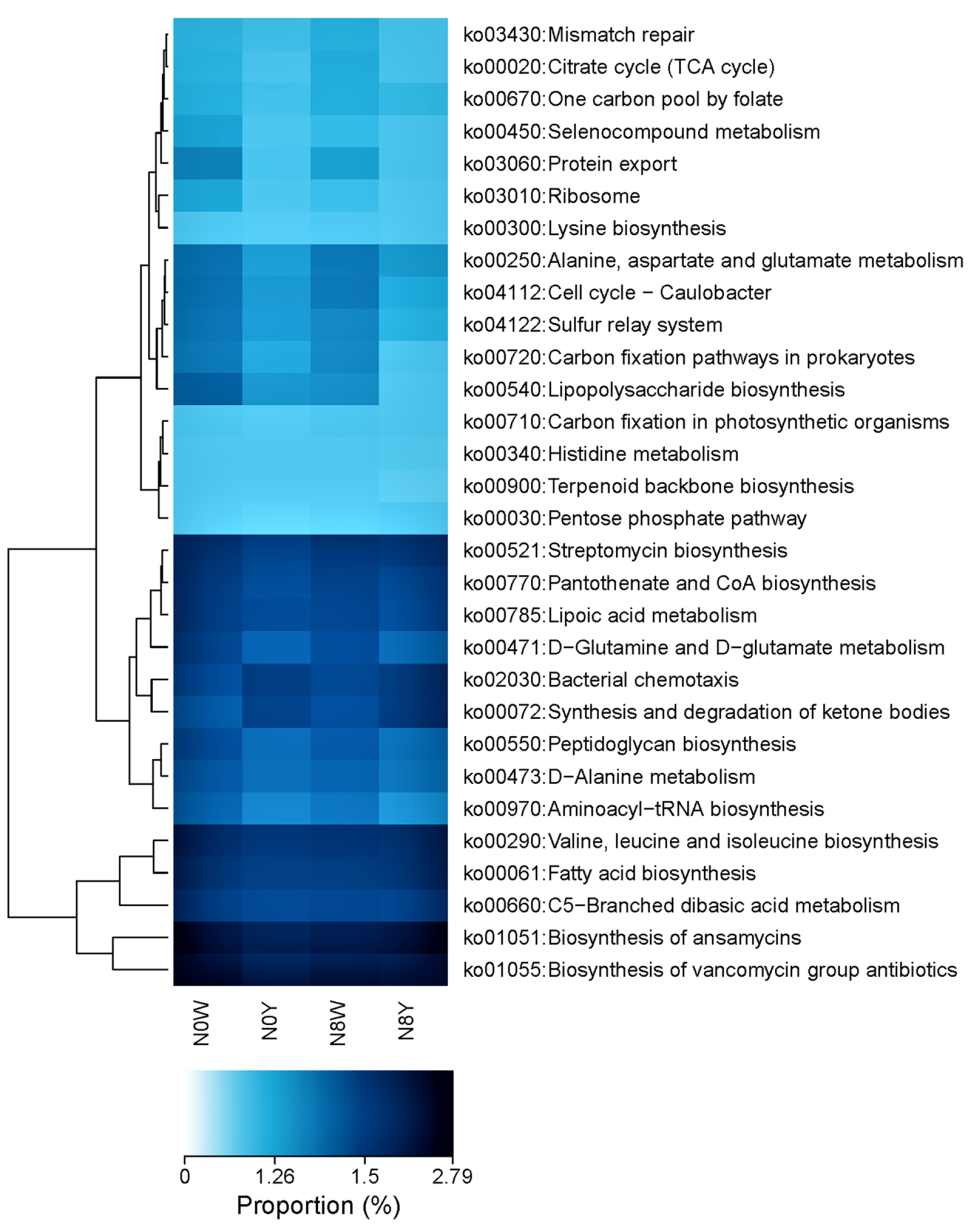
Analysis of microbially mediated KEGG metabolic pathways in sorghum inter-root soil under different nitrogen conditions.

Changes in inter-root soil microbial species and abundance of sorghum roots under different nitrogen conditions caused adaptive changes in soil KEGG metabolic pathways. The KEGG heatmap analysis revealed that mainly 30 pathways changed under N0W, N0Y, N8W, and N8Y four fertilizer patterns. In particular, the ko00540, ko00471, ko02030, ko00072, and ko00550 5 paths changed relatively obviously (Fig. 3). N0Y compared to N0W treatment, and N8W compared to N8Y treatment both exhibited ko02030 (Bacterial chemotaxis) and ko00072 (Synthesis and degradation of ketone bodies) showed significant ratios of the two metabolic pathways, but the level of the difference was more pronounced in N0Y compared to N0W. This indicates that the use of organic fertilizers can increase the metabolic activity of these two pathways, especially at low nitrogen levels. The difference was more significant for N0W compared to N8W in ko00540 (Lipopolysaccharide biosynthesis) pathway and N0Y compared to N8Y in ko00521 (Streptomycin biosynthesis).

### Functional analysis of biological populations involved in soil microsets

**Figure 4:**
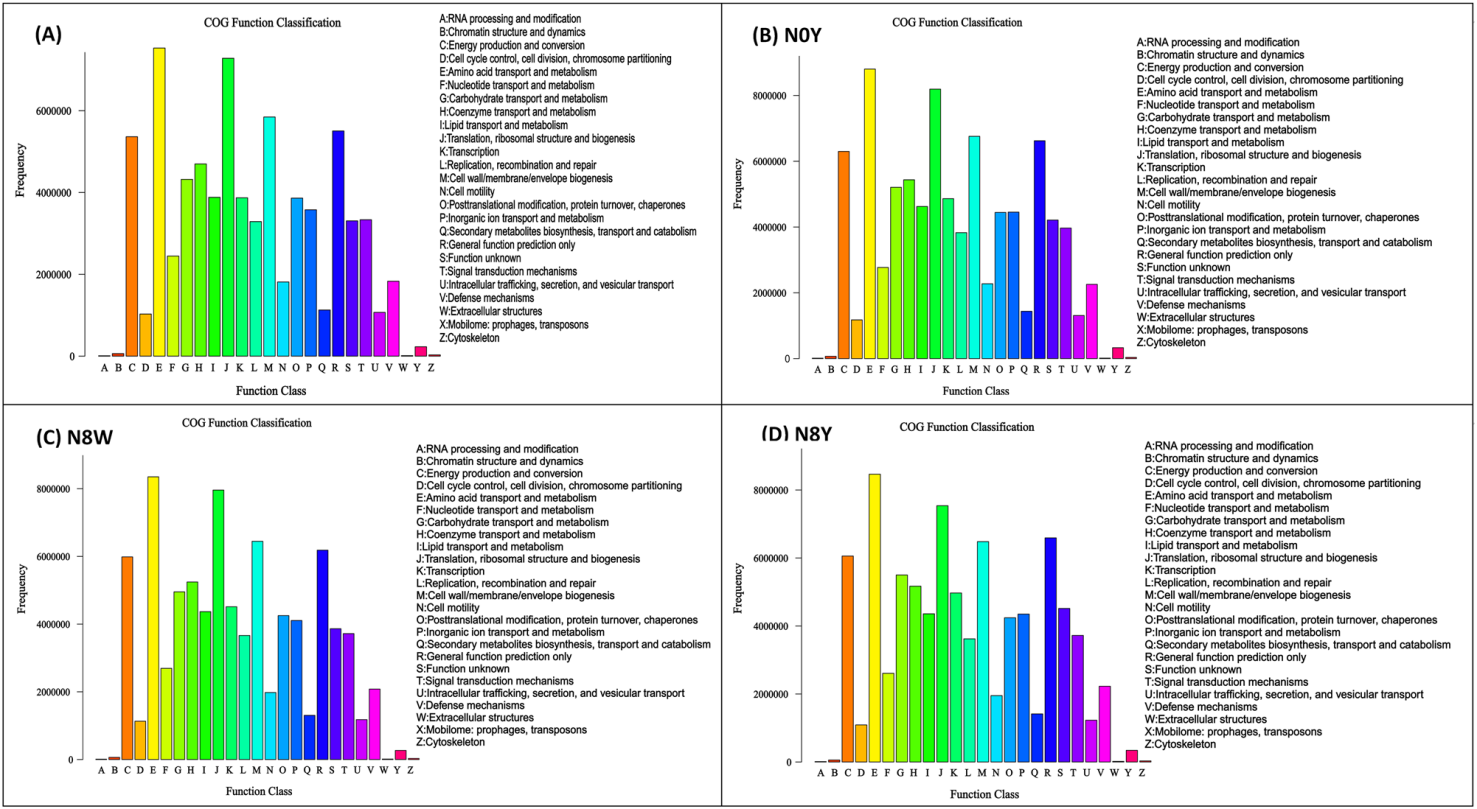
Functional analysis of inter-root soil microorganisms involving biological populations of sorghum under different nitrogen conditions.

There were differences in the function of soil microbial populations between sorghum roots under different nitrogen conditions. It showed that a higher frequency of functional class bacteria in the organic fertilizer treatment than in the non-organic fertilizer treatment at the same level of nitrogen application, i.e. N0Y was higher than N0W ,N8Y was higher than N8W, and the trends of the different functional groups were basically the same (Fig. 4). However, it is noteworthy that intracellular trafficking, secretion, and vesicular transport (U) were significantly lower in N0W compared to N0Y fertilizer pattern. Meanwhile, it is noteworthy that amino acid transport and metabolism (E) appeared significantly more frequently than nucleotide transport and metabolism (F) under both N0 level (N0Y and N0W) treatments, while under N8 level (N8Y The frequency of amino acid transport and metabolism (E) was significantly higher than that of nucleotide transport and metabolism (F) at the N8 level (N8Y and N8W), in contrast to the N0 level.

### Analysis of metacyc pathway involved in soil microbial alterations

The metacyc pathway differed among the four fertilizer application patterns of N0W, N0Y, N8W, and N8Y (Table 2). The frequency of PWY-3781 (aerobic respiration I, cytochrome c) was highest in the different fertilizer application patterns, and N0Y was higher than N0W, while the differences between N8W and N8Y were small at the N8 level. In addition, the frequency of occurrence inosine-5'-phosphate biosynthesis I, UMP biosynthesis, L-histidine biosynthesis, peptidoglycan biosynthesis III (mycobacteria) were relatively in a higher level. In addition, ectoine biosynthesis differed little between N0Y and N0W at the N0 level, while it was significantly greater at N8Y than at N8W. This suggests that ectoine biosynthesis is likely to be the key metabolic pathway regulated by the application of organic fertilizers to soils under moderately high nitrogen conditions.

**Table 2:** Cluster analysis of differential populations of soil microorganisms between sorghum roots under different nitrogen conditions.

Serial number	Number of occurrences (times)				N0W/N0Y%	N8W/N8Y%	Pathway	Pathway description
	N0W	N0Y	N8W	N8Y				
1	2518	4868	3212	3698	51.7	86.8	PWY-6182	Superpathway of salicylate degradation
2	206	368	331	517	56.0	64.1	PWY-5529	Superpathway of bacteriochlorophyll a biosynthesis
3	2305	3490	2877	4328	66.0	66.5	PWY-5705	Allantoin degradation to glyoxylate III
4	22,170	31,305	26,112	30,359	70.8	86.0	LEU-DEG2-PWY	L-leucine degradation I
5	2276	3062	2596	3078	74.3	84.3	P461-PWY	Hexitol fermentation to lactate, formate, ethanol and acetate
6	13,892	18,416	16,431	18,959	75.4	86.7	PWY-181	Photorespiration
7	32,728	40,717	36,758	38,845	80.4	94.6	PWY-6353	Purine nucleotides degradation II (aerobic)
8	26,864	31,922	29,505	29,850	84.2	98.8	HISDEG-PWY	L-histidine degradation I
9	11,465	13,370	12,305	12,660	85.8	97.2	POLYAMSYN-PWY	Superpathway of polyamine biosynthesis I
10	534	608	650	2533	87.7	25.7	PWY-3661	Glycine betaine degradation I
11	449	508	338	572	88.2	59.0	PWY-5531	Chlorophyllide a biosynthesis II (anaerobic)
12	47,460	53,252	50,494	49,557	89.1	101.9	PWY-5695	Urate biosynthesis/inosine 5'-phosphate degradation
13	37,628	41,656	41,592	39,766	90.3	104.6	PANTO-PWY	Phosphopantothenate biosynthesis I
14	42,316	46,834	45,938	42,571	90.4	107.9	POLYISOPRENSYN-PWY	Polyisoprenoid biosynthesis (E. coli)
15	127,395	140,602	140,453	134,730	90.6	104.2	PWY-3781	Aerobic respiration I (cytochrome c)
16	41,810	46,141	45,457	42,069	90.6	108.1	PWY-6123	Inosine-5'-phosphate biosynthesis I
17	50,709	55,682	54,826	50,473	91.1	108.6	PWY-5686	UMP biosynthesis
18	41,661	45,672	45,233	42,552	91.2	106.3	HISTSYN-PWY	L-histidine biosynthesis
19	40,125	43,689	43,689	40,023	91.8	109.2	PWY-6385	Peptidoglycan biosynthesis III (mycobacteria)
20	25,615	27,482	28,691	28,148	93.2	101.9	HOMOSER-METSYN-PWY	L-methionine biosynthesis I
21	25,257	26,951	25,738	26,459	93.7	97.3	PWY-4984	Urea cycle
22	670	485	723	1469	138.1	49.2	P101-PWY	Ectoine biosynthesis

### Sorghum and thousand grain weight correlation with soil microorganisms

**Figure 5:**
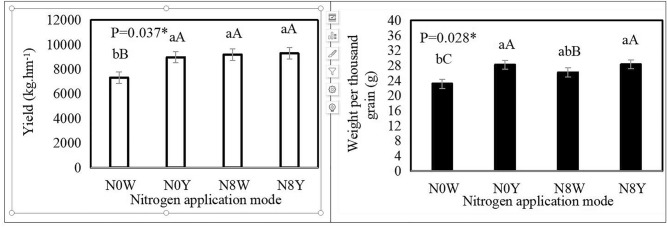
Comparison of yield and thousand grain weight under different nitrogen application patterns. *Lowercase English letters indicates significant difference at 0.05 level, **uppercase English letters indicates significant difference at 0.01 level.

There were significant differences in yield (*P* = 0.037*) and thousand grain weight (*P* = 0.029*) under different fertilizer application patterns (Fig. 5). It is noteworthy that in terms of yield the differences under the three fertilizer application modes N0Y, N8W and N8Y were not significantly higher than N0W, and the trend of thousand grain weight tended to be consistent with yield. The N0Y achieved a higher yield of 8981.4 kg per hm^2^ with organic fertilizers as an alternative to chemical fertilizers, while the analysis revealed that the yield formation of sorghum was closely related to the changes in a large number of soil microorganisms (Table 3), indicating that sorghum under different chemical fertilizers can also achieve higher sorghum yields with a relatively small amount of nitrogen supply combined with the regulation of the activity of inter-root soil microbial populations. The results show that sorghum can also achieve higher yields under different chemical fertilizers through relatively low nitrogen supply combined with regulation of inter-root soil microbial populations.

**Table 3:** Correlation analysis of photosynthetic parameters, yield and thousand grain weight of sorghum with major soil microorganisms.

	Correlation coefficient							
	Chl	Pn	Gs	Ci	Tr	WUE	Thousand grain weight	Yield
Chitinophagaceae	0.71	0.76	0.71	0.59	0.44	0.52	0.94**	0.99 **
Koribacteraceae	0.72	0.69	0.72	0.41	0.26	0.40	0.90**	0.99**
Comamonadaceae	0.82 *	0.86*	0.83 *	0.33	0.29	0.47	0.91**	0.98**
Oxalobacteraceae	0.47	0.52	0.56	0.29	0.51	0.62	0.84**	0.97**
Hyphomicrobiaceae	0.52	0.47	0.49	0.53	0.60	0.25	0.89**	0.96**
Xanthomonadaceae	0.49	0.41	0.37	0.51	0.24	0.47	0.87*	0.96**
Flavobacteriaceae	0.45	0.36	0.44	0.68	0.37	0.42	0.81	0.96**
PRR-10	0.52	0.61	0.49	0.44	0.56	0.32	0.86*	0.95**
Thermoactinomycetaceae	0.75	0.79	0.82*	0.77	0.61	0.41	0.88*	0.95 **
Actinosynnemataceae	0.70	0.76	0.73	0.65	0.63	0.55	0.79	0.95 **
Alteromonadaceae	0.85 *	0.91*	0.93**	0.71	0.49	0.65	0.82*	0.95**
Ellin517	0.78	0.81*	0.79	0.45	0.55	0.71	0.75	0.95 **
Streptosporangiaceae	0.75	0.69	0.72	0.56	0.65	0.26	0.78	0.94**
Sphingomonadaceae	0.85 *	0.89*	0.75	0.78	0.47	0.35	0.72	0.94**
Gaiellaceae Bacillaceae	0.81 *	0.78	0.77	0.71	0.33	0.49	0.81*	0.93 **
Ellin6075	0.75	0.64	0.65	0.62	0.79	0.72	0.74	0.93 **
Solibacteraceae	0.79	0.70	0.63	0.75	0.37	0.78	0.75	0.92 **
Sphingobacteriaceae	0.93**	0.95**	0.89 **	0.82 *	0.63	0.82 *	0.85*	0.92**
Nitrospiraceae	0.91 **	0.92**	0.94 **	0.71	0.51	0.83*	0.81*	0.91 **
Caulobacteraceae	0.89 *	0.92**	0.90**	0.84 *	0.82*	0.48	0.72	0.90 **
Cytophagaceae	0.92**	0.94**	0.89 **	0.68	0.76	0.62	0.85*	0.90**

There are a large number of microbial populations associated with sorghum yield formation, and this table compares 21 groups of bacteria selected for highly significant correlation with yield based on yield and microbial correlation. *Indicates significant difference at 0.05 level, ** indicates significant difference at 0.01 level.

### Sorghum photosynthetic parameters correlated with soil microorganisms

**Figure 6:**
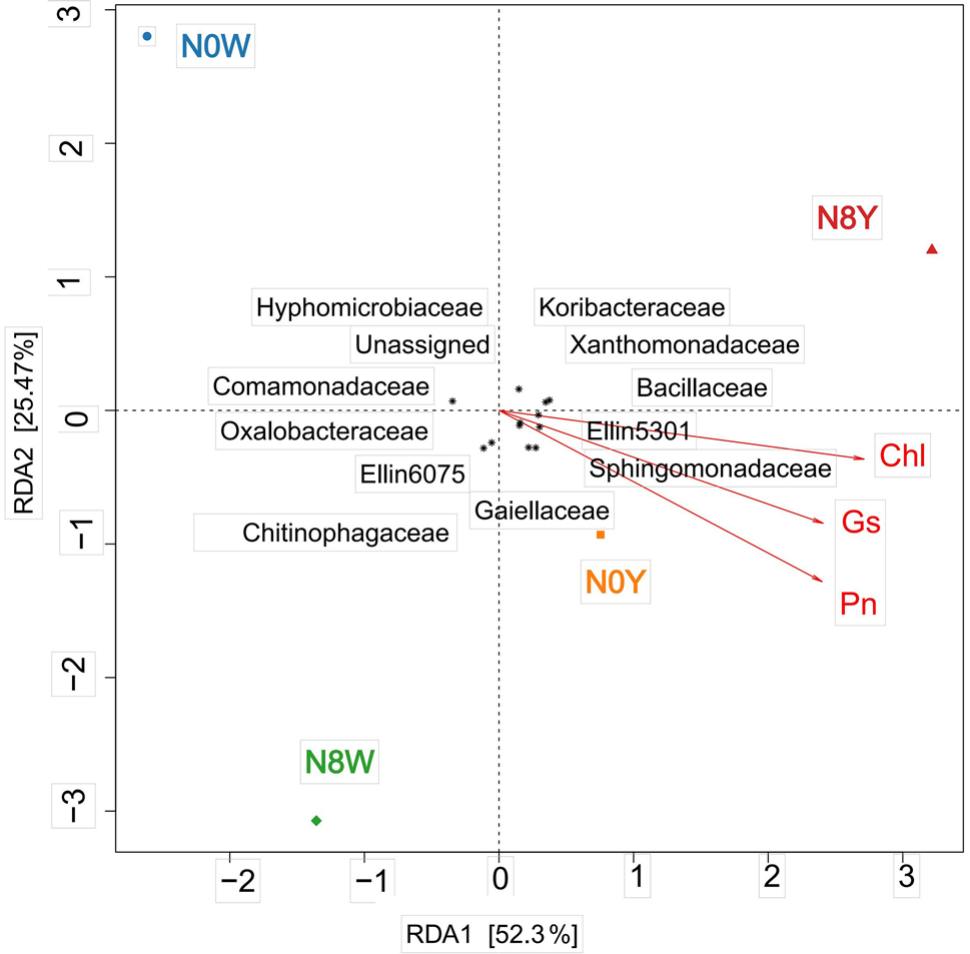
Photosynthesis-related focal microflora of sorghum populations under different nitrogen application conditions.

There were significant effects on net photosynthetic rate (*Pn*), stomatal conductance (*Gs*), intercellular CO_2_ concentration (*Ci*), and transpiration rate (*Tr*) of sorghum under optimal integration of N fertilizer application and organic manure (Table 3). Among them, except for the N0W treatment, the differences in net photosynthetic rates of the other three treatments (N0Y, N8W, and N8Y) were not significant, indicating that additional organic fertilizer and additional nitrogen fertilizer can complement each other. The changes of stomatal conductance and transpiration rate were positively correlated with net photosynthetic rate and the trends were basically the same, while intercellular CO_2_ concentration was negatively correlated with net photosynthetic rate. Also of interest is the close association of soil microorganisms with the photosynthetic process in sorghum (Table 4, Fig. 6), with chitinophagaceae, koribacteraceae, comamonadaceae, oxalobacteraceae, and hyphomicrobiaceae playing a favorable regulation.

**Table 4:** Comparison of photosynthetic parameters of sorghum populations under different nitrogen application conditions.

Nitrogen treatment	Net photosynthetic rate (μmol.m^-2^.s^-1^)	Stomatal conductance (mol H_2_O.m^-2^.s^-1^)	Intercellular CO_2_ concentration (μmol.m^-2^.s^-1^)	Transpiration rate (μmol CO_2_.mol^-1^)	Chlorophyll content (mg.g.FW^-1^)
N0W	18.65b	0.27b	312.7a	4.29b	1.08b
N0Y	25.67a	0.32ab	298.43ab	5.21a	1.51a
N8W	25.91a	0.34a	285.36b	5.33a	1.56a
N8Y	26.16a	0.34a	254.19c	5.29a	1.59a
P-value between nitrogen treatments	0.008**	0.012**	0.036*	0.041*	0.039*
Organic fertilizer P-value	0.027*	0.034*	0.016**	0.028*	0.045*
N*Organic fertilizer intercropping P-value	0.042*	0.053	0.071	0.043*	0.029*

*Indicates significant difference at 0.05 level, ** indicates significant difference at 0.01 level, lowercase English letters indicates significant difference at 0.05 level,

## Discussion

The comprehensive analysis of this study concluded that N0Y, N8W and N8Y can all meet the nitrogen requirements of sorghum and achieve higher yields. However, considering the economic cost of nitrogen and ecological protection, N0Y is the only efficient green planting model to our study that can meet the nitrogen demand of sorghum while achieving ecological protection. This result is in line with the previous study^21^ which suggested that most of the nutrients of organic fertilizer species are in organic state which are difficult to be used directly by the crop, and through the decomposition of microorganisms various nutrients can be released slowly, which in turn provides the nutrients needed for crop growth.

Organic fertilizers have a more comprehensive range of nutrients and can synthesize active substances required by the crop after decomposition. The results of this study suggest that N-efficient use of sorghum can be achieved under the organic fertilizer application of 6000 kg per hm^2^ without chemical fertilizer (N0Y) model for efficient green production of sorghum, pointing out that under this model, soil microorganisms chitinophagaceae, koribacteraceae, comamonadaceae, and it was suggested that the increase in the number of two key populations, comamonadaceae and ellin5301, is the key to the regulation of efficient N use. This type of research has been reported before, and Yang^22^ showed that crops affect soil microbial activity mainly through root secretions, and that soil enzymatic reaction processes are active at the plant root, and it has also been suggested that organic matter provided by organic fertilizer is a soil microorganism that increases the stability of soil enzymes and improves the efficiency of fast-acting N use^23,24^. In addition, it has also been proposed that the peroxidase enzyme activity in the soil is strongly influenced by microorganisms during crop growth, and its toxic effects on plants can be mitigated by increased application of organic fertilizers^25,26^. The results of these studies are in general agreement with the present study, but relatively few studies have been conducted on the populations of nitrogen efficient sorghum inter-root microorganisms.

Soil microorganisms are diverse and participate in the regulation of sorghum growth and development through their specific metabolic pathways. In this study, we identified two key metabolic pathways, bacterial chemotaxis and bynthesis degradation of ketone bodies, that are closely associated with the efficient use of sorghum N by soil microorganisms at the sorghum root level. These two physiological metabolic pathways have not been reported in the analysis of inter-root microbial nitrogen use in crops and may be related to the unique root aeration structure and specific root base secretions of sorghum, or may be due to the interaction between organic fertilizers and sorghum roots. However, although this pathway has not been reported, similar studies have indicated that increasing soil organic matter and reducing nitrogen fertilizer application to some extent can have a positive impact on the functional diversity of soil microbial communities, promoting bacterial chemotaxis and increasing the activity of colonies favoring nitrogen synthesis^27–29^.

The activity of soil microorganisms is closely related to nitrogen uptake by the root system, and favorable for biological populations and efficient nitrogen uptake and utilization pathways are the key to crop growth and development and yield closure. In this study, we found that microbial populations of chitinophagaceae, koribacteraceae, comamonadaceae and oxalobacteraceae contributed to the photosynthetic process and were closely related to yield formation. Such joint analysis of soil microbial activity with photosynthesis and yield is less reported, Richardson^30^ proposed that the transforming effect of wheat inter-root organisms, which are easy to use the soil environment for nitrogen uptake, promote the activity of soil organisms to increase their diversity and facilitate wheat growth and development. Meanwhile, scholars' studies have generally concluded that the application of organic fertilizers can optimize the species and metabolic pathways of inter-root soil microorganisms of crops, improve soil physicochemical properties, and thus promote crop growth^31–33^.

## Conclusion

In conclusion, the N-efficient use of sorghum Liaonian No. 3 in the N0Y model can achieve chemical fertilizer reduction while promoting ecological conservation. Under this model, 17 populations of soil microorganisms, including chitinophagaceae, koribacteraceae, comamonadaceae, oxalobacteraceae, hyphomicrobiaceae, and xanthomonadaceae etc. were changed actively, especially for comamonadaceae and Ellin53012 showed a significant increase in abundance. Changes in microbial populations regulated sorghum growth mainly through ko00540, ko00471, ko02030, ko00072, ko00550 pathways, where ko02030 (Bacterial chemotaxis) and ko00072 (Synthesis and degradation of ketone bodies) are the two most critical metabolic pathways. In terms of microbial population function, intracellular trafficking, secretion, and vesicular transport were significantly reduced in the N0Y fertilizer model, while amino acid transport and metabolism and nucleotide transport and metabolism were changed actively, which were the key factors in the regulation of microbial population function. The integrated regulation of these soil microbial population changes and metabolic pathways could promote photosynthetic processes and nitrogen uptake and utilization of nitrogen efficient sorghum in the N0Y model, leading to the higher yields of 8981.4 kg per hm^2^.
